# Assessing the regional carbon sink with its forming processes- a case study of Liaoning province, China

**DOI:** 10.1038/s41598-018-33401-2

**Published:** 2018-10-11

**Authors:** Xian-Jin Zhu, Han-Qi Zhang, Yan-Ni Gao, Hong Yin, Zhi Chen, Tian-Hong Zhao

**Affiliations:** 1 College of Agronomy, Shenyang Agricultural University, Shenyang, 110866 China; 2 State Key Laboratory of Environmental Criteria and Risk Assessment, Chinese Research Academy of Environmental Sciences, Beijing, 100012 China; 3 Synthesis Research Center of Chinese Ecosystem Research Network, Key Laboratory of Ecosystem Network Observation and Modeling, Institute of Geographic Sciences and Natural Resources Research, Chinese Academy of Sciences, Beijing, 100101 China; 4 College of resources and environment, University of Chinese Academy of Sciences, Beijing, 100049 China

## Abstract

Assessing the regional carbon sink sets the basis of regional carbon management, which involves many measures but has large uncertainties. Carbon sink assessment scheme based on its forming processes (CSF) is a recently proposed measure but repeatly calculates emission from water erosion and ignored human inducing carbon inputs. Therefore, we revised the CSF by calculating the direct outputs from land surface and adding human returned carbon (HC) to the input. The revised CSF thus involved gross primary productivity (GPP), ecosystem respiration (ER), carbon removal from cropland (CRC), emission from reactive carbon (E_RC_), emission from water erosion (E_wat_), and HC, which can be obtained from public data sources. Then the revised CSF was applied to the Liaoning province of China. The estimated carbon input of Liaoning province during 2000–2014 was 114.77 ± 8.41 TgC yr^−1^, while the carbon output was 110.48 ± 8.38 TgC yr^−1^. The difference between input and output induced a carbon sink of 4.30 ± 2.20 TgC yr^−1^, accounting for 3.75% of total carbon input. The carbon sink spatially decreased from northeast to southwest, which was highly correlated with that of GPP. However, though its forming fluxes significantly increased from 2000 to 2014, the carbon sink showed a decreasing trend. In addition, the revised scheme only needed published and public data, which made it serve as an alternative approach for regional carbon budget assessment.

## Introduction

Carbon sink, defined as the residue of organic matter sequestrated by vegetation photosynthesis after natural and human disturbance^1^, plays an important role in regulating atmosphere CO_2_ concentration^2^. Increasing the carbon sink of terrestrial ecosystems has been regarded as an important approach in mitigating climate change, which needs fully assess the spatial distributions of carbon sink and its increasing potential^3^.

Many approaches, such as the resource inventory^4^, process models^5,6^, and atmospheric inversion^7,8^, were involved in assessing the regional carbon sink. Resource inventory assessed the regional carbon sink as the changes of carbon stocks during different periods, which was much accurate and was deemed as the base of other measures^1,4,9^. However, resource inventory had a coarse temporal resolution and only accounted the value of carbon sink, which can’t directly reveal the processes how carbon sink was formed. Process models quantified regional carbon sink by simulating the carbon cycles with climatic and biotic drivers, which can simulate the carbon sink at different time-scale and can address the forming processes of carbon sink, while its parameters had some uncertainties^10,11^. Atmosphere inversion calculated the regional carbon sink by analysing the changes of atmosphere CO_2_ concentration and other carbon emissions from natural and human activities, which was much simple but strongly relied on the accuracy of other carbon emissions such as land use changes^8,12^. In addition, most of those measures differed obviously as the definitions of carbon sink varied among approaches. For example, resource inventory defined carbon sink as the changes of carbon stock during a period, which ignored the carbon stored in human-beings^13^, while carbon sink in atmospheric inversion reflected the net carbon fluxes including not only the terrestrial ecosystems but also the human beings^12,14^. It is thus necessary to build an assessment scheme fully accounting the involved carbon fluxes, which would improve the clarity of carbon sink they assessed.

Carbon sink assessment scheme based on its forming processes (CSF), a recently proposed approach, involved various carbon fluxes during the forming processes of carbon sink and served as an alternative approach in quantifying the regional carbon sink^15^. However, the current version of CSF had some shortages, which can be ascribed into two aspects. First, it involved many fluxes that may be repeatedly calculated. For example, the carbon emission from water erosion (E_wat_) may be recalculated by the carbon flowing to the ocean. Second, gross primary productivity (GPP) was regarded as the sole input, while there were other input fluxes such as the manure, the straw return and industrial fertilizer (IF) introducing carbon to the ecosystems. Therefore, it was urgent to clearly define the carbon sink and fully considering the involved carbon inputs, which would be helpful for accurately assessing the regional carbon budget.

In this study, selecting Liaoning province as the target, we revised the CSF by calculating the direct outputs from land surface and adding the carbon input by human returned carbon (HC). Then the revised scheme was applied to assess the spatial distributions of carbon sink and its components. Our results will provide a reference for assessing the regional carbon sink in other regions, which will be helpful for reducing the uncertainties in regional carbon sink assessments. Our results will also provide a data basis for carbon management in Liaoning province, whose carbon sink had substantial uncertainties and was sensitive to climate change^5^.

## Results

### The amounts of carbon sink and its forming fluxes

According to data used in this study, we found that the total carbon input of Liaoning province during 2000–2014 was 114.77 ± 8.41 TgC yr^−1^. The carbon input were primarily comprised of GPP, which was 110.78 ± 7.87 TgC yr^−1^ and accounted for 95.62% of total carbon input (Table 1). Another carbon input as HC was only 4.00 ± 0.74 TgC yr^−1^, accounting for less than 4% of total carbon input.

**Table 1:** The amounts of carbon sink and its forming fluxes in Liaoning province of China during 2000–2014.

	Carbon fluxes	Amount (TgC yr^−1^)	Portion (%)	Method
Input	GPP	110.78 ± 7.87	96.52	MODIS
	HC	4.00 ± 0.74	3.48	CRC * 0.2
Output	ER	86.96 ± 5.35	75.76	Eq. (1)
	CRC	19.98 ± 3.72	17.41	^30^
	E_RC_	0.97	0.85	^52^
	E_wat_	2.57	2.23	^16^
Net value	Carbon Sink	4.30 ± 2.20	3.75	Input-Output

Note: GPP and HC were the abbreviations of gross primary productivity and human returned carbon, respectively. ER, CRC, ERC, and Ewat were the abbreviations of ecosystem respiration, carbon removal from cropland, emission from reactive carbon, and emission from water erosion, respectively. Portion was calculated as the ratio of each flux to the total carbon input, which was the sum of GPP and HC. MODIS was the abbreviation of Moderate Resolution Imaging Spectroradiometer data.

The total carbon output during the studied period was 110.48 ± 8.38 TgC yr^−1^ but was comprised of many fluxes (Table 1). Ecosystem respiration (ER) was the largest output, with a value of 86.96 ± 5.35 TgC yr^−1^ accounting for 75.76% of carbon input. Carbon removal from cropland (CRC) also occupied a large portion of output carbon flux, with a value of 19.98 ± 3.72 TgC yr^−1^ accounting for 17.41% of carbon input. However, the amounts of emission from reactive carbon (E_RC_) and emission from water erosion (E_wat_) were relative small, which were 0.97 TgC yr^−1^ and 2.57 TgC yr^−1^, respectively, and accounted for 0.85% and 2.23% of carbon input, respectively (Table 1).

After accounting for the input and output fluxes, the carbon sink of Liaoning province was 4.30 ± 2.20 TgC yr^−1^ and occupied 3.75% of carbon input.

### The spatial distributions of carbon sink and its forming fluxes

**Figure 1:**
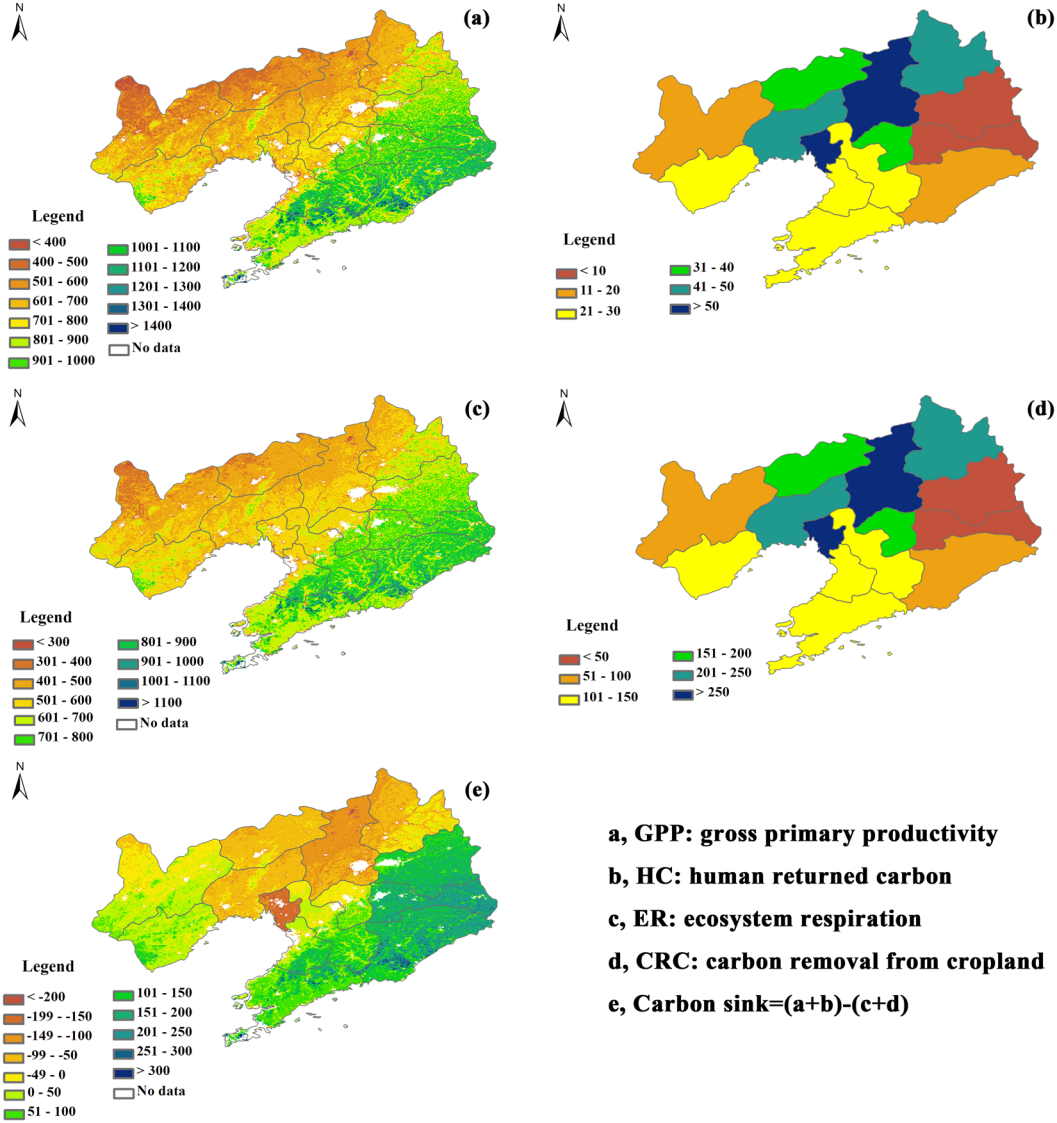
The spatial distributions of carbon sink (**e**) and its forming fluxes, including gross primary productivity (GPP, **a**), human returned carbon (HC, **b**), ecosystem respiration (ER, **c**), and carbon removal from cropland (CRC, **d**), emission from reactive carbon (E_RC_), and emission from water erosion (E_wat_), in Liaoning province of China during 2000–2014. E_RC_ and E_wat_ were not shown as they were deemed to have no spatial variations in this study. The maps were made by ArcGIS 10.0 software (http://www.esri.com/software/arcgis).

The magnitudes of carbon sink and its forming fluxes spatially varied (Fig. 1) but their spatial distributions differed among fluxes.

The input carbon fluxes included GPP and HC. The magnitude of GPP ranged from 73 to 1942 gC m^−2^ yr^−1^, with a decreasing trend from northeast to southwest. The eastern region had a higher value of GPP, which may exceed 900 gC m^−2^ yr^−1^, while the GPP values of western region were lower than 700 gC m^−2^ yr^−1^ (Fig. 1a). As another input carbon flux, HC was small and ranged from 5 to 71 gC m^−2^ yr^−1^ (Fig. 1b). In addition, the spatial distribution of HC showed a decreasing trend from the centre to the border (Fig. 1b), which significantly differed from that of GPP with a correlation coefficient of −0.82 (p < 0.01).

The output carbon fluxes include ER, CRC, E_RC_, and E_wat_. ER spatial varied ranging from 130 to 1300 gC m^−2^ yr^−1^, which was smaller than that of GPP but was the largest output flux. Though the value of ER differed from that of GPP, its spatial distribution also showed a decreasing trend from northeast to southwest (Fig. 1c). CRC also spatially varied with a range of 28 to 353 gC m^−2^ yr^−1^, serving as the second large output carbon flux. The spatial distribution of CRC exhibited a decreasing trend from the centre to the border, with the western region having a higher CRC than the eastern region (Fig. 1d). Given little effort was paid to the spatial variation of E_RC_ and E_wat_ in Liaoning province, we had no choice but supposed E_RC_ and E_wat_ were evenly distributed, with the values of 6.63 and 17.50 gC m^−2^ yr^−1^, respectively.

After integrating the input and output carbon fluxes, we found that the magnitude of carbon sink spatially varied and ranged from −360 to 400 gC m^−2^ yr^−1^. The spatial distribution of carbon sink decreased from east to west, with the highest values appearing at the northeast, while the central region had the lowest carbon sink, which showed a net carbon release. The western region showed a moderate carbon sink or source (Fig. 1e). In addition, the spatial distribution of carbon sink was similar to that of GPP and ER (r = 0.86, p < 0.01) but showed an opposite trend with that of CRC and HC (r = −0.42, p < 0.01).

### The inter-annual variations of carbon sink and its forming fluxes

**Figure 2:**
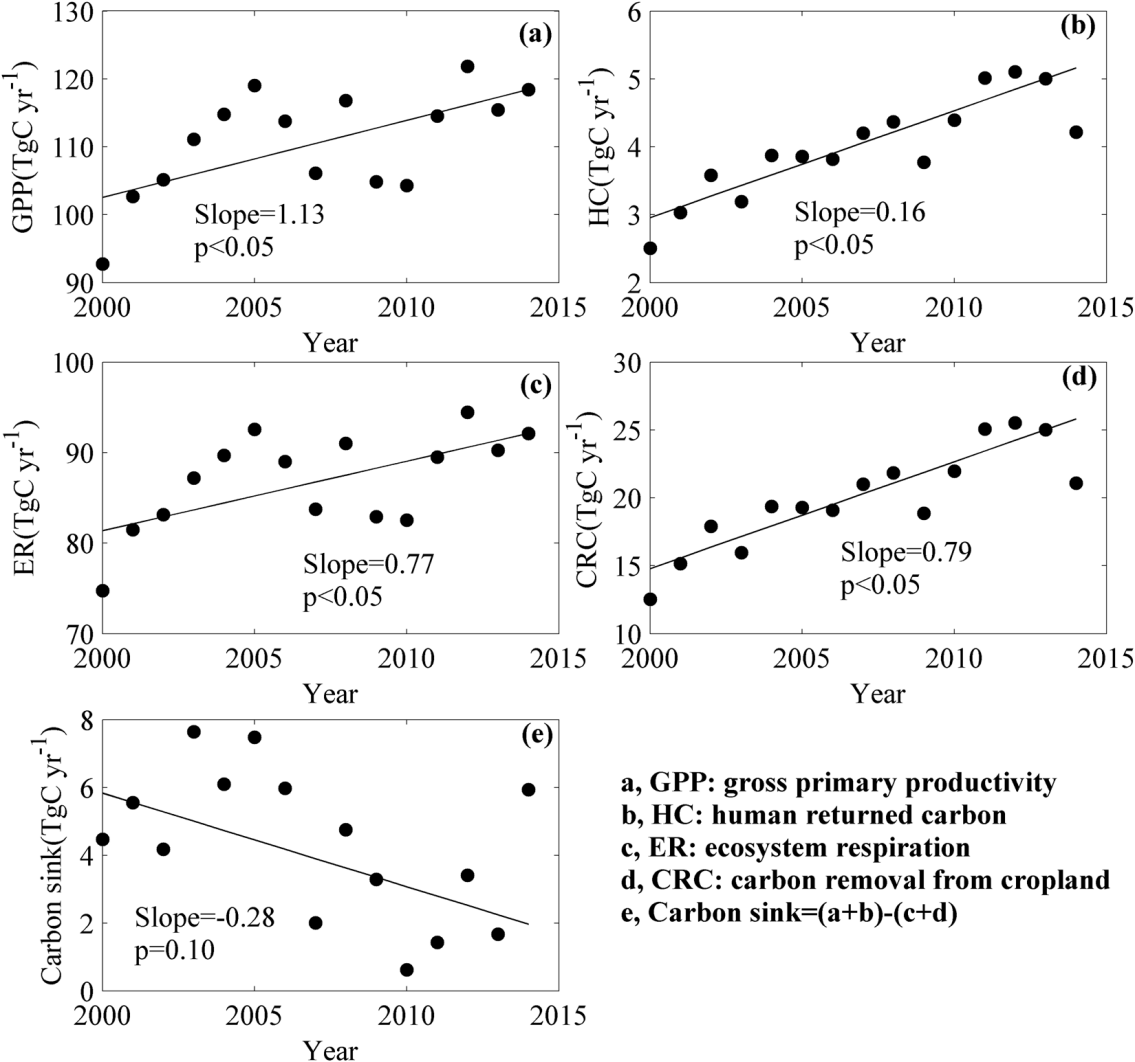
The inter-annual variations of carbon sink (**e**) and its forming fluxes, including gross primary productivity (GPP, **a**), human returned carbon (HC, **b**), ecosystem respiration (ER, **c**), and carbon removal from cropland (CRC, **d**), emission from reactive carbon (E_RC_), and emisssion from water erosion (E_wat_), in Liaoning province of China during 2000–2014. E_RC_ and E_wat_ were not shown as they were deemed to have no inter-annual variations in this study.

Besides their spatial variations, carbon sink and its forming fluxes exhibited significant inter-annual variations (Fig. 2), which differed among fluxes.

The input carbon fluxes showed significant increasing trends during 2000–2014 (Fig. 2a,b). From 2000 to 2014, GPP, ranging from 92.77 to 121.85 TgC yr^−1^, significantly increased with an increasing rate of 1.13 TgC yr^−1^ (Fig. 2a). HC, ranging from 2.51 to 5.11 TgC yr^−1^, also exhibited a significant increasing trend, with an increasing rate of 0.16 TgC yr^−1^ (Fig. 2b).

Significant increasing trends were also found in output carbon fluxes (Fig. 2c,d). ER ranged from 74.73 to 94.48 TgC yr^−1^, with an increasing rate of 0.77 TgC yr^−1^ (Fig. 2c), while CRC ranged from 12.53 to 25.52 TgC yr^−1^, with an increasing rate of 0.79 TgC yr^−1^ (Fig. 2d).

Though the input and output carbon fluxes exhibited significant increasing trends, the increasing rate of the input carbon fluxes was smaller than that of the outputs (Fig. 2). Therefore, carbon sink, the net carbon budget between input and output, showed a significant decreasing trend with a rate of 0.28 TgC yr^−1^ (Fig. 2e).

## Discussion

In this study, we revised the CSF and applied it to Liaoning province of China to analyse the spatial and temporal variations of carbon sink from 2000 to 2014. Comparing with its original version^15^, the revised CSF was modified in three aspects. First, HC was added as another input carbon flux as it was an important component of carbon inputs (Table 1). Second, the net carbon emission caused by water erosion was considered in the revised CSF. After suffering from water erosion, the eroded soil carbon experienced some processes such as the denudation, deposition, and exchange, which made the eroded soil carbon be partly deposited in ecosystems^16^. Third, the carbon flux flowing to the ocean from major rivers was deleted as it had been calculated as the carbon emission from water erosion.

However, we didn’t consider the carbon emissions from fires, diseases, pests, and rats, which may be ascribed as two reasons. First, carbon emissions from fires, diseases, pests, and rats were smaller as they were seldom occurred in recent years in this region^15,17^. Second, data about these emissions were seldom reported in this region. Therefore, the revised CSF was applicable in regions like Liaoning province, while if it was applied to regions having much natural disturbances like fires, carbon emissions from such disturbances should be incorporated into the revised CSF.

Furthermore, owning to the simplicity of the revised CSF, there were some uncertainties for the forming fluxes thus the carbon sink, which needed to be addressed in the future.

1. Though it had a higher spatial resolution^18^, the used GPP had some uncertainties, which may primarily be ascribed into two aspects. First, the spatial variations of model parameters in generating GPP were not clearly considered. For example, the light use efficiency model used the fixed parameters in each vegetation type^19^, while the parameters substantially varied within each vegetation type^20,21^. Second, the used GPP had a poor capacity in sequestrating the inter-annual variation of GPP^22,23^, which was also true for other models. Therefore, the effects of climate and biotic factors, especially the frequently occurred extreme events^24,25^, on the inter-annual variation of GPP may not be able to be fully captured.
2. The calculation of ER was simple but had some uncertainties. In this study, we calculated ER from the empirical relationship between GPP and ER^26^, which was feasible in theory^27^ and simple to be applied. However, the empirical relationship may vary among regions and years^28,29^, especially when the regions suffered from extreme climate events.
3. The calculation of CRC was simple but had a coarse spatial resolution. We calculated CRC from the yield and harvest index, which was very simple as the yield can be found in the statistical yearbooks^30^, while the statistical yearbooks only reported the yield at each prefectural level city, which made the spatial resolution of CRC was very poor. In addition, harvest index may vary among regions^31^, which may further introduce some uncertainties to CRC.
4. HC calculation may be oversimplified and may have some uncertainties. In this study, we calculated HC as CRC multiplying a constant coefficient (0.2), while the returning coefficient may differ among regions. If we assumed the returning coefficient randomly varied from 0 to 0.5 in each prefectural level city, which was randomly repeated 500 times, the uncertainty represented by the standard deviation of HC also spatially varied (Fig. 3). The uncertainty ranged from 3.87 to 46.94 gC m^−2^ yr^−1^, showing a decreasing trend from centre to the border. The uncertainty of HC magnitude induced an uncertainty of HC ranging from 1.87 to 8.13 TgC yr^−1^. The uncertainty of HC accounted for no more than 3.60% of the total carbon input, which may make some effects on the main results of this study.

**Figure 3:**
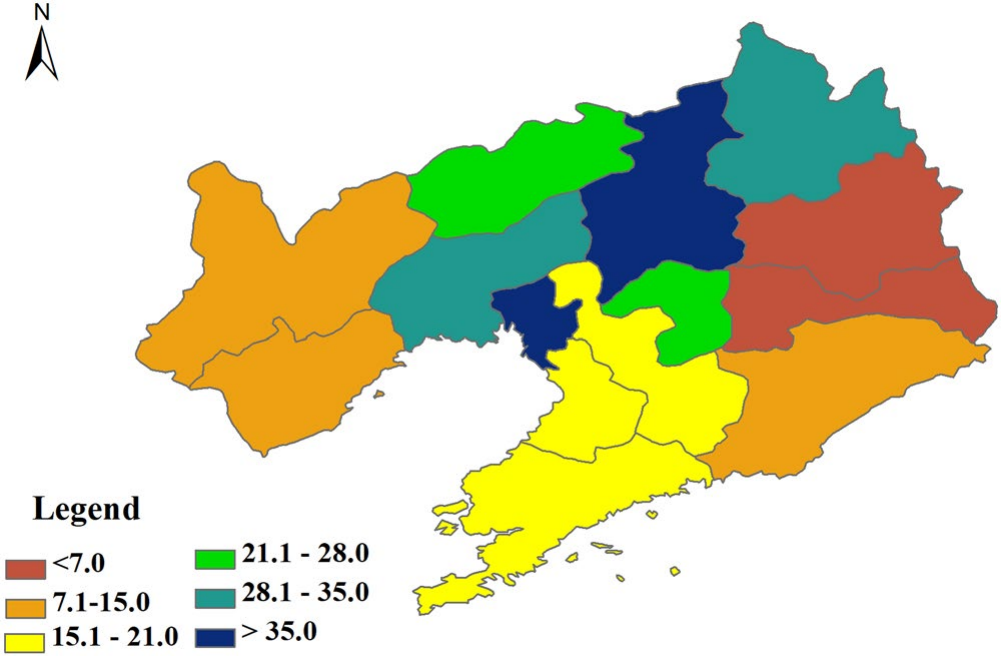
The uncertainty of human returned carbon (HC) in Liaoning province of China. The uncertainty was represented by the standard deviation of randomly calculated HC, which assumed the returning coefficient randomly varied from 0 to 0.5 in each prefectural level city and randomly repeated 500 times. The maps were made by ArcGIS 10.0 software (http://www.esri.com/software/arcgis).
5. The spatial and inter-annual variations of E_RC_ and E_wat_ were not available. Though we attempted to included E_RC_ and E_wat_ in the revised CSF, there was little effort in quantifying their spatial and inter-annual variations in this region, which made the derived carbon sink have some uncertainties.

Though there were some uncertainties in the revised CSF, it only relied on public data, which made it simple to be applied in regions having no meteorological or inventory data. In addition, the carbon sink in the revised CSF was coincided with the original definition of carbon sink as the residual organic carbon in terrestrial ecosystems^1^. Furthermore, we also found that the amount of HC (4.00 TgC yr^−1^) was comparable with the sum of E_RC_ (0.97 TgC yr^−1^) and E_wat_ (2.57 TgC yr^−1^) (Table 1), indicating that it was feasible for ignoring the HC, E_RC_, and E_wat_ in current models in assessing the regional carbon budget.

**Figure 4:**
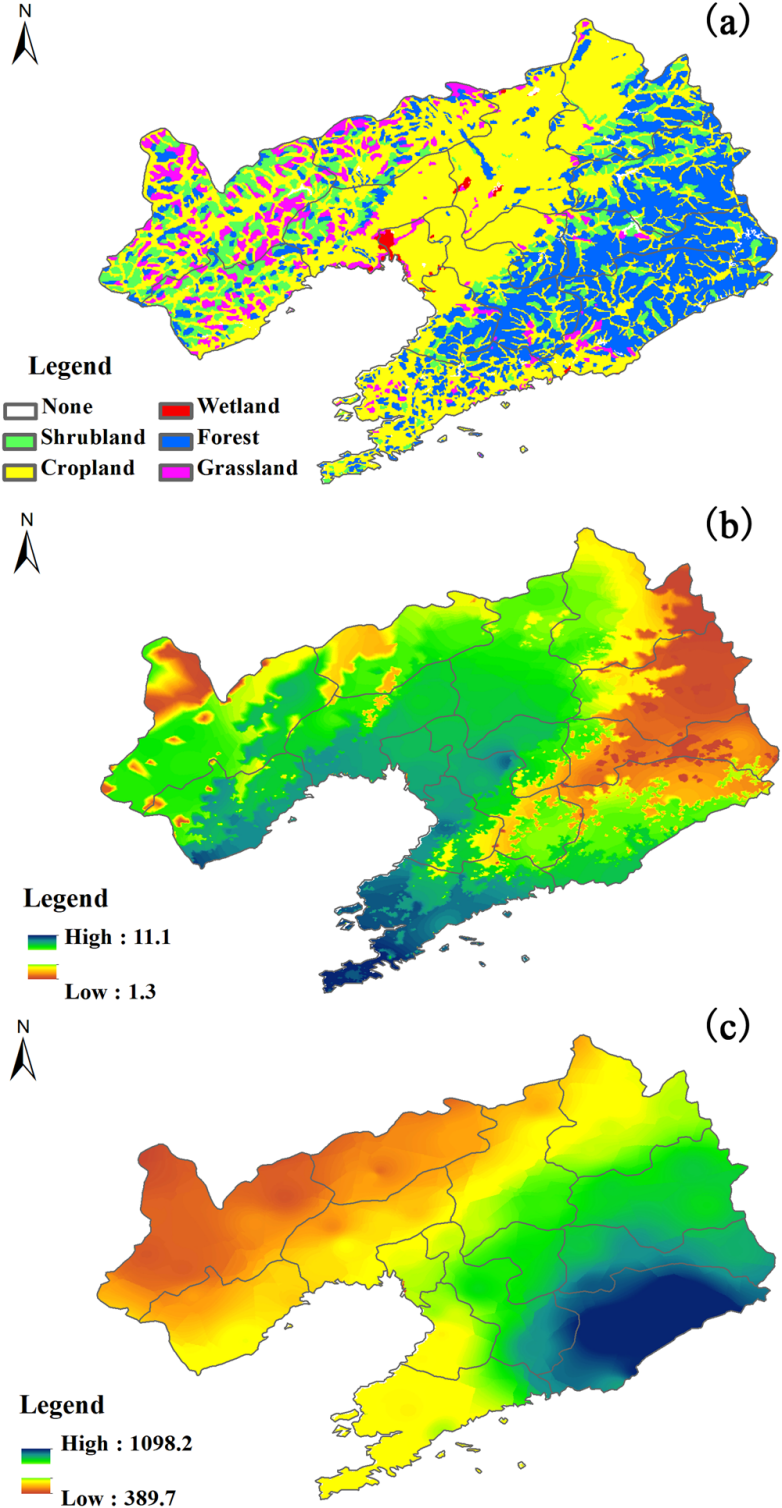
The spatial distributions of ecosystem type (**a**), annual mean air temperature (**b**), and annual precipitation (**c**) in Liaoning province of China from 2000 to 2014. The maps were made by ArcGIS 10.0 software (http://www.esri.com/software/arcgis) based on our previous data^36^.

Based on the revised CSF, we found that GPP showed a decreasing trend from northeast to southwest (Fig. 1a) while CRC showed decreasing trend from the centre to the border (Fig. 1d), which made carbon sink decrease from east to west. In addition, all forming fluxes such as GPP, HC, ER, and CRC exhibited significant increasing trends, while carbon sink decreased from 2000 to 2014. The spatial distributions of carbon sink and its forming fluxes may be related to the spatial variations of climate and vegetation (Fig. 4). Annual mean air temperature (MAT) and annual precipitation (MAP), which dominated the spatial variation of GPP^21,26,32,33^, showed decreasing trends from south to the north (Fig. 4b) and from east to the west (Fig. 4c), respectively. The spatial distributions of MAT and MAP, especially MAP, jointly determined that of GPP as water was the limiting factor in this region, which made GPP decrease from northeast to southwest (Fig. 1a). However, the spatial distribution of CRC was primarily affected by the ratio of planting area to the land area^30^, which was higher in regions with much cropland. The spatial distributions of ecosystem type showed that the centre of this region was mostly occupied by cropland (Fig. 4a), which made CRC higher in the centre. Therefore, CRC showed a decreasing trend from the centre to the border (Fig. 1d). The spatial distributions of GPP, CRC, ER, and HC joint determined that of carbon sink. Among years, CRC significantly increased (Fig. 2d) as more fertilizer was used, which also made GPP increase from 2000 to 2014 (Fig. 2a). However, the increasing rate of ER and CRC was higher than that of GPP and HC, which made carbon sink show a decrease trend in this period (Fig. 2e).

## Conclusions

In this study, we revised the carbon sink assessment scheme and applied it to Liaoning province of China. The revised scheme only involved gross primary productivity (GPP), ecosystem respiration (ER), carbon removal from cropland (CRC), human returned carbon (HC), emissions from reactive carbon (E_RC_), and emissions from water erosion (E_wat_), which relied on public and published data. The estimated input and output were 114.77 ± 8.41 TgC yr^−1^ and 110.48 ± 8.38 TgC yr^−1^, which made a carbon sink of 4.30 ± 2.20 TgC yr^−1^. Carbon sink spatially decreased from northeast to southwest and positively correlated with GPP. Though its forming fluxes significantly increased, carbon sink decreased from 2000 to 2014. The revised scheme was very simple thus provided an alternative approach to assess the regional carbon budget.

## Methods

### The theoretical basis of carbon sink assessment scheme

**Figure 5:**
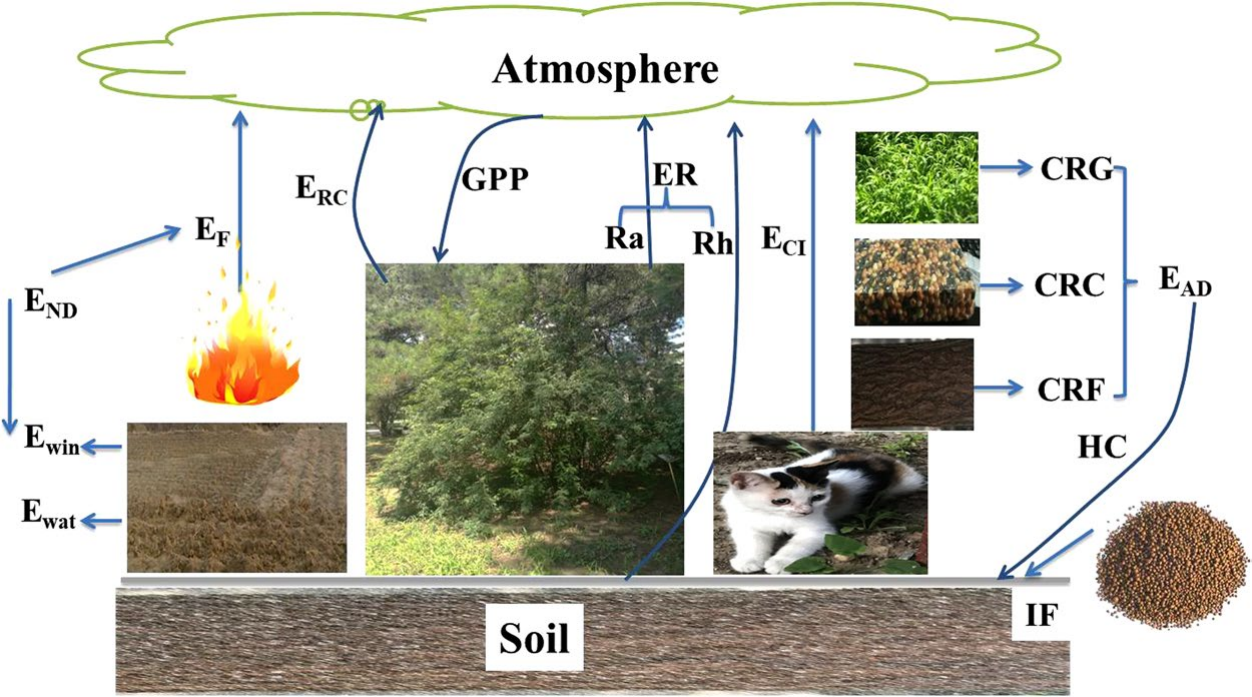
Carbon cycle processes in terrestrial ecosystems. The abbreviations of items used in the figure were as below: GPP (gross primary productivity), Ra (autotrophic respiration), Rh (Heterotrophic respiration), ER (ecosystem respiration), E_RC_ (emission from reactive carbon), E_CI_ (emission from creature ingestion), CRC (carbon removal from cropland), CRG (carbon removal from grassland), CRF (carbon removal from forest), E_F_ (emission from fire), E_win_ (emission from wind erosion), E_wat_ (emission from water erosion), E_AD_ (emission from anthropogenic disturbances), E_ND_ (emission from natural disturbances), HC (human returned carbon), IF (industrial fertilizer). The figure was made with Microsoft Powerpoint 2010 (https://products.office.com/en-us/powerpoint). All individual images were taken by ourselves.

The theoretical basis of carbon sink assessment scheme was the carbon cycle processes in terrestrial ecosystems (Fig. 5)^15,34^. Carbon cycle in terrestrial ecosystems started from GPP, which was the first step in sequestrating CO_2_ from the atmosphere. The sequestrated carbon was firstly released by plants through autotrophic respiration (Ra). Then the remained organic carbon, which was deemed as the net primary productivity (NPP), was further consumed by animal and microbes through heterotrophic respiration (Rh). Ra and Rh constructed to ER. Furthermore, the sequestrated carbon was consumed by creature ingestion, which can be deemed as the emission from creature ingestion (E_CI_). During the stages that ecosystem released carbon through CO_2_, the sequestrated carbon was also released by reactive carbon such as CH_4_, CO, and so on, which was deemed as the emission from reactive carbon (E_RC_). Besides the natural releases, the sequestrated carbon also suffered from natural disturbances and human disturbances. Emissions from natural disturbances (E_ND_) was caused by fire (E_F_) and soil erosion, where E_ND_ from soil erosion can be further separated into wind erosion (E_win_) and water erosion (E_wat_). Emissions from anthropogenic disturbances (E_AD_) were comprised by the carbon removal from cropland (CRC), grassland (CRG), and forest (CRF). After human disturbances, some removed carbon can be returned to the ecosystems as human returned carbon (HC). In addition, industrial fertilizer (IF) such as ammonium bicarbonate (NH_4_HCO_3_), urea (CO(NH_2_)_2_) also took some carbon into the ecosystems.

### The revision of carbon sink assessment scheme based on its forming processes

**Figure 6:**
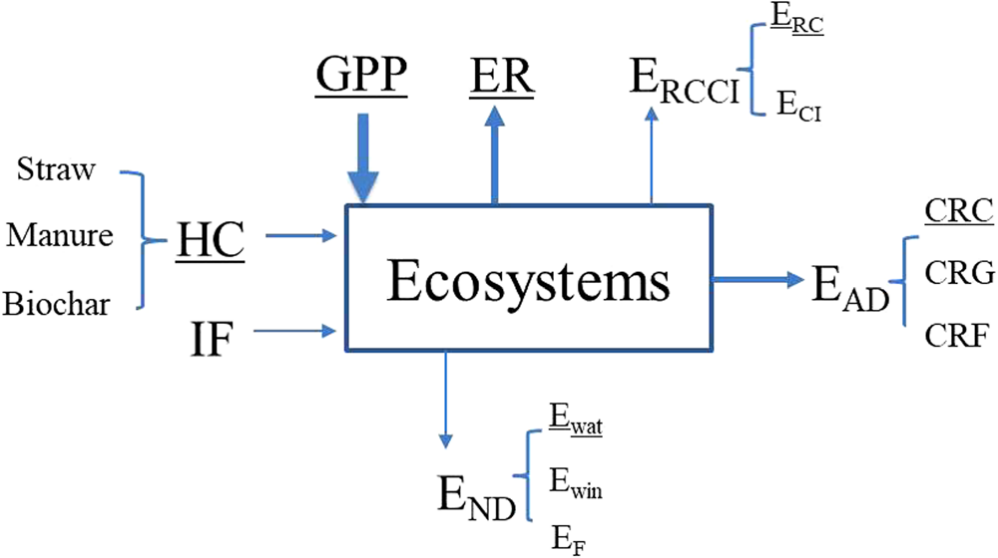
Carbon fluxes involving in calculating the regional carbon sink based on its forming processes. The input carbon fluxes were gross primary productivity (GPP), industrial fertilizer (IF), and human returned carbon (HC), which further includes returning from straw, manure, and biochar. The output fluxes included ecosystem respiration (ER), emission from reactive carbon and creature ingestion (E_RCCI_), emission from anthropogenic disturbances (E_AD_), and emission from natural disturbances (E_ND_). E_RCCI_ were comprised by emission from reactive carbon (E_RC_) and creature ingestion (E_CI_). EAD was the carbon removal from cropland (CRC), grassland (CRG), and forests (CRF), while E_ND_ was caused by fire (E_F_) and soil erosion from water (E_wat_) and wind (E_win_). The fluxes with underlines were used in this study.

Carbon sink was traditionally defined as the residual carbon in terrestrial ecosystems^1,34^. Therefore, the carbon sink assessment scheme based on its forming processes involved carbon fluxes occurred between the ecosystems and the surrounding environments. According to the theoretical basis of carbon sink assessment scheme, the input carbon fluxes were GPP, HC, and IF, while the output carbon fluxes included ER, E_RCCI_, E_AD_, and E_ND_ (Fig. 6).

Given the low carbon content of IF, which can be calculated from their chemical formula such as ammonium bicarbonate (NH_4_HCO_3_), urea (CO(NH_2_)_2_), we only considered GPP and HC as the input carbon fluxes. In addition, given E_CI_ occupied a small proportion of GPP^15^ and Liaoning province was dominated by cropland, whose E_CI_ can be neglected, we set E_CI_ to 0 in this region. Furthermore, E_AD_ was primarily comprised by cropland carbon transfer in this region and the carbon removal from grassland and forest were small but were hard to obtain^35^, we just had CRC as E_AD_. Given fire seldom occurred and soil erosion by wind was hardly quantified in this province, we only considered emissions from water erosion (E_wat_) as the emissions of natural disturbance (E_ND_).

Therefore, the carbon fluxes involved in calculating carbon sink of Liaoning Province were GPP, HC, Ra, Rh, E_RC_, CRC, E_wat_, which was indicated by the underlines of carbon fluxes in Fig. 6.

### Calculating the magnitudes of carbon fluxes

The magnitudes of some carbon fluxes, including GPP and ER, which had detailed spatial distributions, were calculated by each grid. The magnitude of GPP was set to the Moderate Resolution Imaging Spectroradiometer data (MOD17A2) having a spatial resolution of 30 arc second from University of Montana (http://www.ntsg.umt.edu/project/modis/mod17.php). Given GPP and ER were positively spatially coupled^26,36^, ER, whose spatial resolution was same as GPP, was calculated from GPP as:

1 $${\rm{ER}}=0.68\,{\rm{GPP}}+81.90$$

The magnitudes of some carbon fluxes, including CRC and HC having limited information on their spatial distribution, were calculated by each prefectural level city from statistical yearbooks^37–51^. CRC was calculated from the yields of agricultural products (*Y*_i_), the crop harvest index (*HI*_i_), the water content (*C*_wi_) and the carbon fraction of dry matter (*C*_Ci_), which can be found in our previous work^30^. The amount of HC was set to 20% of CRC.

While the magnitudes of other carbon fluxes, such as E_RC_ and E_wat_, whose spatial distributions were seldom available, were calculated as the ratio of their total amounts to the regional area. The amounts of these carbon fluxes can be calculated from the following subsection.

The magnitude of carbon sink was calculated from the input and output fluxes, with a spatial resolution of 30 arc second.

### Calculating the amounts of carbon fluxes

The amounts of GPP and ER, were summed from the magnitude of carbon fluxes by each grid, while these of CRC and HC were calculated as the sum of their corresponding fluxes in each prefectural level city, which can be found in our previous work^30^.

Emission from reactive carbon included that from methane (CH_4_), nonmethane volatile organic compounds (NMVOC) and carbon monoxide (CO)^15^. Emission from CH_4_ (E_m_) occurred at rice paddies, lakes, and natural wetlands^52^. From a recently published paper^52^, we can get the values of E_m_ of Liaoning province from rice paddies and natural lake. In addition, E_m_ from natural wetland was comparable with that from natural lake^52^. Therefore, E_m_ of Liaoning province was estimated from previous work^52^ by summarizing the E_m_ from rice paddies, natural lake, and natural wetland. Given little attention was paid to the spatial variation of NMVOC and CO emissions in China, we had no choice but calculated the emissions of NMVOC (E_NM_) and CO (E_CO_) of Liaoning province from the total emissions of China^15^ and the proportion of Liaoning land area in Chinese area. E_RC_ was the sum of E_m_, E_NM_, and E_CO_.

Water erosion caused the lateral and vertical transportations of soil carbon^16^. The lateral transportation included the carbon removal (F1) and the deposition of eroded carbon (F2), while the vertical transportation included the replacement of atmosphere CO_2_ (F3), the decomposed carbon from buried and transported carbon to atmosphere (F4 and F5). The net lateral and vertical transportations of eroded carbon were F1-F2 and F3-F4-F5, respectively. E_wat_ was the sum of net lateral and vertical transportations with a positive value indicating the carbon emission. In a recent paper, E_wat_ of northeast China was reported by Yue, *et al*.^16^. After integrating the area of three northeast provinces and E_wat_ of northeast China, we estimated emissions of soil water erosion in LiaoninNg province.

### Data analysis

In this study, we analysed the spatiotemporal variations of carbon sink and its components during 2000–2014. The data of GPP, ER, CRC, and HC were calculated from 2000 to 2014 by each year. However, given the values of E_RC_ and E_wat_ were smaller and were hardly obtained, their inter-annual variations were neglected.

Based on data from 2000 to 2014, we calculated their mean values during the study period to investigate their spatial distributions. The spatial correlations between fluxes were conducted with ArcGIS 10.0 software (http://www.esri.com/software/arcgis), with α of 0.05.

Using the Manna-Kendall trend analysis, we detected the trend of each carbon fluxes from 2000 to 2014, which was conducted with Matlab 2014.

## Data Availability

The datasets analysed during the current study are calculated based on the methods described in this study and the original data of the statistical yearbook, which can be found at www.cnki.net. All datasets generated during the current study are available from the corresponding author on reasonable request.
